# Lesson Learned from Impact of COVID-19 Pandemic on People with Visual Impairment

**DOI:** 10.31662/jmaj.2023-0193

**Published:** 2023-12-27

**Authors:** Kimiko Ueda

**Affiliations:** 1 Faculty of Health and Well-being, Kansai University, Osaka, Japan

**Keywords:** visual impairment, COVID-19 pandemic, difficulty in daily life

The COVID-19 pandemic affected the physical and mental health of the entire population ^(1), (2)^ and limited their lives and activities, especially for people with disabilities ^(3)^. The people with visual impairment (VI), estimated over 349 million worldwide ^(4)^, touch, tractile contact, and act upon objects every day. Exploring the difficulties caused by COVID-19 for people with VI will help us understand the criticality of the challenges they face. It brings into sharp relief the difficulties of people with VI before the COVID-19 pandemic.

To understand the overview of the COVID-19 challenges on people with VI, we have to be aware of its impact on individuals across the spectrum of visual impairment ^(5)^. The letter of formal request from the Japan Federation of the Visually Impaired, the largest organization of people with VI in Japan, is useful. It summarized the support needed for various daily problems reported by members: priority distribution of necessary hygiene items (masks and rubbing alcohol) to people with VI and their caregivers; bailout program for people employing acupuncture, moxibustion, and massages; guarantee and continuity of accompanying support system; provision of appropriate information to people with VI; appropriate information distribution and supports for people with VI on examination and treatment of COVID-19; tailored supports for children enrolled in special support schools. Hand hygiene was more important than usual for people with VI who understand using their hands. Accompanying supports are a guarantee of action and information for people with VI. However, due to the COVID-19 pandemic, offices providing accompanying support were closed and the number of guide helpers decreased, restricting in their lives. Information about COVID-19 changed day by day. Information from the national and local governments, dealers regarding changes in business hours, infection countermeasures, and so on made it difficult for people with VI to obtain information timely and appropriately, causing difficulties and anxiety among them. Regarding various support programs from the national and local governments such as special fixed benefits, sustainability benefits, and livelihood welfare fund loan programs, people with VI experienced difficulties in obtaining information about these support programs. Even if information was obtained, filling out the application forms was challenging. In many cases, welfare services such as accompanying supports, in-home nursing care, and substitute writing/reading support were required to prepare the application forms. Because these supports directly affected the lives of people with VI, the national and local governments needed to take responsibility for the notification and application of these supports.

The sound environment is important for the daily routine of people with VI ^(6)^; however, COVID-19 greatly affected the sound environment, which in turn had a significant impact on their daily lives. The main cues used by people with VI to walk and act alone are information from a white cane, sound, airflow, and smell. Contextually, sound, airflow, and smell information were considerably affected by COVID-19. People with VI use sound instead of sight to move and confirm their current position. A change in environment, such as the absence or reduction of the usual sounds and voices, considerably affects their daily lives. When they walk, they can recognize turns by the flow of air, but wearing masks made it difficult to perceive the flow of air. Additionally, the change in daily air flow due to open doors (including automatic doors) for ventilation had a great impact on their daily lives. The same occurred for smells.

Considering these backgrounds, the COVID-19 pandemic impacted the quality of life and daily activities, as well as mental health ^(7)^. Ito et al focused on these conditions after the COVID-19 pandemic ^(8)^. In a cross-sectional survey using a questionnaire, they reported that even after the COVID-19 pandemic, the perceived daily difficulties of people with VI were associated with health-related quality of life. Despite the limitation that the study was not comparative with the prepandemic situation and has a selection bias, results suggested that people with VI might be affected by the COVID-19 pandemic longer than those without VI and take longer to return to ordinal life postpandemic. Further research is needed in the future.

Even before the COVID-19 pandemic, people with VI faced various challenges and poor health-related quality of life in their daily lives, caused by low accessibility of information, prejudice, and lack of understanding ^(9)^. The pandemic served an opportunity to focus on their difficulties in life and be widely recognized. Notably, people with VI are vulnerable in emergencies, and more adequate supports should be provided and further understanding be achieved even in ordinary times.

## Article Information

### Conflicts of Interest

None
